# Culturally Tailored Women’s Empowerment Strategies to Improve Immunization Uptake: Formative Research Using a Human-Centered Design Approach

**DOI:** 10.2196/60089

**Published:** 2025-10-28

**Authors:** Muyi Aina, Uchenna Igbokwe, Raihanah Ibrahim, Itoro Ata, Safiya Atta, Tobiloba Adaramati, Kunle Oreagba, Ernest Ezeogu, Precious Ojo Uahomo, Eric Aigbogun Jr

**Affiliations:** 1 Solina Centre for International Development and Research (SCIDaR), 8 Libreville Crescent, Wuse II, Abuja, 900288, Nigeria; 2 Department of Anatomy, Faculty of Basic Medical Sciences, Enugu State University of Science and Technology, Enugu, Nigeria

**Keywords:** routine immunization, female caregivers, human-centered design, focus group discussions, key informant interviews, Northern Nigeria

## Abstract

**Background:**

Human-centered design (HCD) is vital for crafting impactful and successful health interventions. This approach has been effective in enhancing the uptake of health services, especially in rural areas. The Routine Immunization Buddy System (RIBS) is an HCD-driven intervention designed to support economically disadvantaged caregivers by empowering them to take greater responsibility for their own health and the immunization of their children.

**Objective:**

Through a community-based participatory research (CBPR), this study seeks to understand the economic, social, and health lifestyles and the impact on the health-seeking attributes of the potential beneficiaries of the project. This ensures that the identified intervention and its strategy will be implemented in a way that is of utmost benefit to the users.

**Methods:**

Using a 3-step HCD process—discovery, ideation, and formulation—the study conducted focus group discussions and key informant interviews for caregivers and stakeholders capable of influencing the woman’s decision within a health setting. Semistructured interviews facilitated an in-depth exploration of participants’ experiences. Data collection used audio and video recordings, detailed notes, and observation sessions. Data analysis encompassed transcription, descriptive analysis, and thematic analysis to inform evidence-based interventions.

**Results:**

Four key interconnected barriers to routine immunization were identified from the formative research: (1) vaccine hesitancy, which is driven by safety concerns and inconsistency in vaccine-related messaging; (2) gender-based decision-making constraints, where male approval often influenced access to care; (3) financial limitations affecting transport and medical costs; and (4) unequal access to health care due to geographic disparities. Caregivers emphasized the need for economic empowerment and trusted community-based health education. These findings informed a critical redesign of the intervention from a 2-arm to a 3-arm model, with peer support groups and financial empowerment components added to address both economic barriers and vaccine misinformation through culturally tailored approaches.

**Conclusions:**

The use of HCD-informed formative research has helped identify tailored women’s empowerment strategies, such as economic support, community-led advocacy, and peer networks, that show promise in addressing health disparities among caregivers. These approaches are hoped to enhance caregivers’ ability to make informed health decisions, ultimately contributing to improved immunization uptake and broader community well-being. The insights from this research are actively informing the design of our upcoming cluster randomized controlled trial, which aims to strengthen women’s decision-making power around immunization services.

## Introduction

Immunization is an essential and cost-effective public health strategy that reduces childhood morbidity and mortality, saving an estimated 2-3 million lives each year [1]. Despite its proven benefits, routine immunization (RI) coverage in Nigeria has remained persistently low. A key measure of RI system performance is the coverage of the third dose of the pentavalent vaccine, commonly referred to as Penta-3. This vaccine protects against 5 serious childhood diseases—diphtheria, pertussis (whooping cough), tetanus, hepatitis B, and *Haemophilus influenzae* type b (Hib)—and is typically administered at 14 weeks of age [2]. In Nigeria, the Penta-3 coverage rate stands at 50%, which indicates that RI coverage has consistently lagged [3]. Particularly concerning is the disparity observed across regions, with Northern Nigeria shouldering the heaviest burden of unimmunized children [4,5]. In the North Central, North East, and North West zones, Penta-3 coverage rates are alarmingly low, at 54%, 37%, and 29%, respectively [3]. These findings underscore the urgent need for targeted interventions aimed at improving RI uptake in Northern Nigeria to mitigate the risks posed by vaccine-preventable diseases and safeguard the health of vulnerable populations.

In contrast, the South-East, South-South, and South-Western zones have higher Penta-3 coverage rates of 83%, 70%, and 74%, respectively. Vaccination coverage was found to improve across the geopolitical zones with increasing mothers’ wealth and education levels [2]. The results from the second quarter (Q2) of 2019 Lot Quality Assurance Sampling (LQAS, a methodology used to assess the quality and coverage of health interventions) and the Performance Assessment for Performance Management (PAPA, a framework for evaluating and improving program implementation) further supported these conclusions [3,6-9].

Community involvement, which means creating a cooperative partnership between communities and immunization programs, is highlighted in the World Health Organization’s 2016 report on Global Routine Immunization Strategies and Practices (GRISP) as 1 of the 9 crucial investments to attain improved immunization results [10]. Human-centered design (HCD) is an effective approach to fostering community involvement and ownership. Five HCD strategies can be seamlessly integrated into community-based participatory research (CBPR) to enhance results: establish transdisciplinary teams, prioritize empathy, engage and collaborate with “extreme users,” swiftly prototype, and develop tangible products or services [11]. HCD enables the attainment of a deep understanding of the problem a solution aims to address and a comprehensive grasp of its target users, thus serving as the cornerstone of impactful solutions [12,13]. HCD involves cultivating profound empathy for individuals, uncovering their needs and desires, and crafting customized interventions to address their challenges [14,15]. Only through this comprehensive understanding can a product be developed that resonates with users [16-19].

Despite ongoing efforts to increase immunization rates, Nigeria is still home to an estimated 3.1 million of the world’s unvaccinated children [18]. Demand creation for vaccination remains a critical component of national immunization programs. It ensures that parents, caregivers, communities, and stakeholders understand the importance of immunization, believe in the safety and efficacy of vaccines, trust the quality and reliability of health services, and possess both the knowledge and motivation to complete the immunization schedule [20]. Establishing sustainable demand for immunization depends not only on community trust in the health system but also on the ability of caregivers (primarily women) to access, afford, and act upon timely health information [21,22].

In Northern Nigeria, where social and cultural norms often limit women’s autonomy in health care decision-making, empowering women is particularly vital. When women are equipped with the right knowledge, financial resources, and decision-making power, they are more likely to seek and complete immunization services for their children. This formative research was therefore designed to understand the lived realities of women in the intervention communities, identify the key social and structural determinants that influence health-seeking behaviors, and codevelop innovative, culturally appropriate strategies to overcome these barriers. The study delineated targeted goals across key thematic areas and clarified contextual challenges and user needs to inform the design of a women’s empowerment-focused randomized controlled trial aimed at enhancing RI uptake in the region.

The Routine Immunization Buddy System (RIBS) initiative is designed to empower unemployed mothers in Nigeria by integrating vaccination education with vocational skills training to enhance RI coverage. The program specifically targets rural communities with historically low immunization rates and a high prevalence of unemployed women, aiming to reduce children’s vulnerability to vaccine-preventable diseases. RIBS establishes a direct linkage between community health systems, the local structures and networks that deliver primary health services, including immunization, maternal, and child health interventions, and economic empowerment initiatives, which are structured programs that provide individuals with income-generating skills, tools, and opportunities to improve their financial independence. Through this linkage, RIBS leverages both health education and livelihood development to drive behavior change.

## Methods

### The RIBS Framework

Under the RIBS framework, participating mothers are organized into small support groups and paired with one another as accountability partners. Group leaders are trained by community health workers to deliver practical, culturally relevant information about vaccines using a visual teaching aid called the *Hannun Rigakafi* (Immunization Hand). In parallel with this health education, participants are introduced to potential employment pathways, such as small-scale farming or trade, and receive corresponding tools and training.

This integrated approach strengthens mothers’ confidence and motivation to complete the recommended 5 essential childhood immunizations [23]. The RIBS program was initially piloted in Kaduna State, Nigeria, and included an embedded evaluation component to assess improvements in immunization knowledge and demand among participating mothers.

### Formative Research Approach

#### Research Design

This study was a formative study that used an HCD [24] aimed at designing a women empowerment program to enhance immunization service use through qualitative data gathering [25]. This method was used to gather insights directly from the target population to inform the development of the program, ensuring that cultural nuances, community perspectives, and social determinants are considered in designing the intervention. Through collaboration with diverse stakeholders, including the head of the house, community leaders, and health care professionals, the study aims to develop culturally sensitive and contextually relevant interventions. This process will be useful in modifying an existing intervention or designing a better one that will be tailored to address the disparities in health care access and use, ultimately enhancing immunization coverage and improving health care outcomes within the target community.

**Figure 1.**
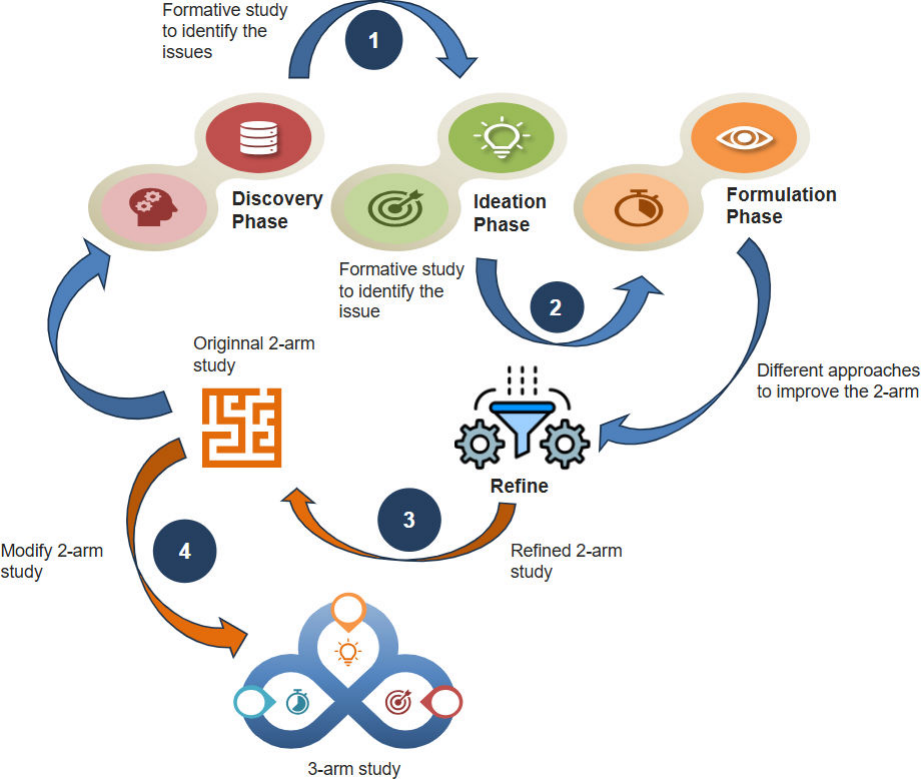
Overview of the study design and participant selection process for the cluster randomized controlled trial.

The HCD approach involved a 3-phase HCD process**—**discovery, ideation, and formulation**—**followed by the remodification of the original randomized controlled trial study concept, as illustrated in Figure 1.

#### Discovery Phase: Understanding the Lives and Experiences of Caregivers in the Community

##### Approach

This phase used a CBPR [26] to provide a comprehensive understanding of the lives and experiences of caregivers in Kaduna State, with particular focus on the challenges they faced, resulting in limited access to health care services and RI uptake. Recruitment and data collection were conducted during this discovery phase, engaging with local stakeholders, including community leaders, religious figures, and caregivers, to ensure their active involvement in the research process in understanding and addressing immunization challenges effectively [27,28].

##### Study Setting and Population

The study was conducted in a semiurban and a rural area, specifically targeting communities with distinct religious compositions—one predominantly Christian and the other Muslim. This selection allowed for a holistic contextual understanding of the role of culture and religion in describing the specific socioeconomic barriers affecting caregivers in seeking immunization services. Two wards—Kachia and Angwan Yelwan Makaranta—were selected from Kachia and Soba local government areas (LGAs), respectively, in Kaduna State. Kachia is a semiurban settlement with a mix of Christian and Muslim populations, primarily engaged in farming activities such as ginger, yam, maize, and millet cultivation. Angwan Yelwan Makaranta, on the other hand, is a rural settlement predominantly inhabited by Muslims, with farming being the main occupation for men. Participants include caregivers (specifically stay-at-home mothers and other female primary caregivers) of children aged under 5 years who expressed willingness to participate in the study.

##### Participant Recruitment

Eligible participants were identified through a community-based recruitment strategy involving local health workers and leaders. Caregiver inclusion criteria included (1) being a primary female caregiver aged 18‐40 years with at least one child under 12 months, (2) not being in formal employment, (3) being a current resident in the study communities, and (4) willingness to participate in group discussions. Community stakeholders (religious leaders, male household heads, and women’s group leaders) were selected based on their formal or informal leadership positions and influence on health-seeking behaviors. The female caregivers were identified through household visits, while religious leaders, male decision makers, and women’s group leaders were invited through community networks. The 3-week recruitment process included sensitization meetings and written informed consent, with all participants assigned confidential identifications. Trusted community intermediaries facilitated culturally appropriate enrollment to ensure diverse representation.

##### Research Instruments Design

For the data collection, a sequential approach of focus group discussions (FGDs) and then key informant interviews (KIIs) was adopted. The semistructured FGD guide incorporated an adapted version of the 5C scale, assessing psychological antecedents of vaccination such as confidence, complacency, constraints, calculation, and collective responsibility [29]. This framework guided discussions to uncover barriers and contextual factors influencing immunization uptake, offering a nuanced understanding of caregivers’ perceptions and behaviors. This KII guide was developed to follow through with the FGDs for the mothers, exploring issues around women empowerment, financial autonomy, decision-making in immunization, and collective agreement in seeking health. Both tools were structured to include an introduction, demographic information, open-ended questions, probing questions, follow-up questions, a closing message, consent, and confidentiality assurances. Additionally, the tool enabled gathering information about the social, economic, and health status of community caregivers, including their receptiveness to health interventions involving peer support and economic empowerment.

##### Data Collection

FGDs were strategically tailored to engage caregivers, facilitating dynamic group interactions that encouraged the sharing of collective experiences and perspectives. These discussions provided a valuable space for participants to delve into common challenges and insights related to seeking health care, particularly for mothers. Conversely, KIIs were designed to tap into the understanding of community stakeholders who play pivotal roles in shaping maternal health-seeking behaviors. These interviews offered deeper insights into the intricate dynamics of community influences on health care decisions (Table 1).

FGDs and KIIs were conducted at the Primary Health Center (PHC) Soba in Soba LGA, PHC Kachia, and PHC Gumel in Kachia LGA. This was facilitated by the community health extension workers, community volunteers, and community leaders. The data collection included audio and video recordings of the interview processes. Although the questionnaires were prepared in English, interviews were conducted in the preferred dialects of the participants, which were either Hausa or English. To safeguard confidentiality and integrity, robust data management protocols were implemented, ensuring secure storage and systematic organization of the collected information. Data collection was conducted over 3 days.

**Table 1.** The sample frame for the qualitative approach.

Study participants	FGDs^a^ (n=7)	Individuals per FGD (n=24)	KIIs^b^ (n=2)
Caregivers	4	7	—^c^
Stakeholders			
Husbands	1	7	—
Religious leaders	1	5	—
Community leaders	1	5	—
Community-women leader	—	—	1
Market women leader	—	—	1

a FGDs: focus group discussions.

b KIIs: key informant interviews.

c Not applicable.

##### Data Analysis

The plan for data analysis involved the transcription of audio and video recordings from FGDs and KIIs. The transcriptions were coded into relevant themes and analyzed using the grounded theory approach for thematic analyses [30,31]. This involved 2 research team members forming concepts from the data and independently identifying several themes. The researchers agreed upon the themes and coded open-ended comments for each theme. We evaluated each comment using the constant comparative method of grounded theory [31].

### Ideation Phase: Creative and Collaborative Process of Generating Ideas and Solutions

The ideation phase of the study began with brainstorming sessions and collaborative discussions among the research team, caregivers, and community stakeholders. This phase aimed to devise a comprehensive intervention that resonated with the needs and aspirations of caregivers and stakeholders while effectively addressing the research objectives. Various ideas were explored, taking into account factors such as community dynamics, resource availability, and feasibility. Insights were synthesized, and themes were drawn from the results obtained from the discovery phase.

### Formulation Phase: Changing Concepts Into Tangible and Achievable Interventions

In the formulation phase of the HCD process, insights and identified opportunity areas were translated into concrete design concepts. This phase focused on brainstorming ideas, refining concepts based on responses from caregivers and stakeholders, and developing solutions that address the needs and challenges identified during the discovery phase. Collaborative cocreation sessions were conducted to engage stakeholders and ensure that the proposed solutions align with the preferences and aspirations of the caregivers.

### Ethical Considerations

This study was reviewed and approved by the ethics research committee of the Department of Planning, Research, and Statistics, Kaduna State Ministry of Health (MOH/ADM/744/VOL.1/601). The committee reviewed and approved the study protocol, data collection instruments, and informed consent documents prior to the commencement of the research. Written informed consent was obtained from all study participants prior to their participation. Consent forms were made available and administered in English, Hausa, or Arabic, depending on the participant’s preference. Participants were informed of the study’s purpose, procedures, potential risks and benefits, and their right to withdraw at any point without consequence.

To protect participant privacy and confidentiality, all data were anonymized at the point of transcription. Audio and video recordings were securely stored in password-protected folders, and personal identifiers were removed during transcription. Only authorized members of the research team had access to the data. Participants received a modest token of appreciation to compensate for their time, valued at approximately 2000 Nigerian Naira (approximately US $2.5) in the form of basic household items or transportation reimbursement. No images of identifiable individuals are included in this manuscript or supplementary materials. Should such materials be included in future dissemination, written consent for publication of identifiable images will be obtained and archived in accordance with ethical standards.

## Results

### Grounded Theory Analysis

The formative phase of the HCD approach yielded rich insights into the community’s context, which were organized into themes using grounded theory analysis. Table 2 summarizes the key themes and corresponding findings. Notably, an additional theme related to vaccination perceptions and barriers emerged, highlighting caregivers’ challenges and concerns about immunization.

**Table 2.** Themes from the grounded theory analysis of the interviews.

Themes	Findings
Sociocultural context	Soba and Kachia illustrated how religious diversity influences social norms and cultural behaviors among caregivers.
Education and economic status	Early pregnancy and marriage significantly increase the rate of school dropouts among girls, subsequently limiting their access to further education and economic empowerment opportunities.
Health care access and use	Caregivers recognized the importance of accessing health care for the well-being of themselves and their families. However, they encountered various obstacles that hindered their ability to obtain and use health care services effectively.
Family dynamics and decision-making	The study uncovered notable differences in marital dynamics among caregivers, emphasizing variations in spousal support, extramarital affairs, and decision-making autonomy across different communities.
Future aspirations and community development	Caregivers expressed a strong desire for economic empowerment as a means to enhance their families’ standard of living and financial stability.
Vaccination perceptions and barriers	Concerns about vaccine safety, misinformation, and logistical challenges hinder access to immunization services and contribute to low routine immunization uptake.

### Understanding the Lives and Experiences of Caregivers in the Community

#### Sociocultural and Economic Dynamics

The study revealed diverse sociocultural influences, educational challenges, health care access barriers, varied family dynamics, and a strong desire for economic empowerment among caregivers in Soba and Kachia communities. These findings provide valuable insights into the multifaceted challenges and aspirations within the communities of Soba and Kachia, highlighting the need for targeted interventions that address sociocultural, economic, and health care–related issues to promote holistic community development.

#### Sociocultural Context

Caregivers in Soba and Kachia communities demonstrated how religious variety affects social norms and cultural behaviors. These communities differ in how women are viewed, empowered, and how much autonomy they have in making decisions.

*I only interact with other women at social gatherings such as weddings and naming ceremonies.*

[A caregiver from Soba]

#### Education and Economic Status

Early pregnancy and marriage greatly raised the percentage of school dropouts among girls, which subsequently limited their access to further education and economic empowerment. For both sexes, agriculture served as the primary source of income, with women actively engaged in small-scale farming and trade.

*My academic journey came to an end when I got pregnant in senior secondary school.*

[A caregiver from Kachia]

#### Health Care Access and Use

Caregivers acknowledged that access to health care is critical to preserving and advancing their own and their family’s health and well-being. Nonetheless, caregivers faced several obstacles that made it difficult for them to get and make use of health care services. Financial limitations were the main cause of these obstacles, keeping many caregivers from getting necessary prescriptions or obtaining prompt medical attention. Fear of adverse effects after vaccination, lack of supplies and medications at the hospital, nonconsenting spouses, and competing religious practices are some of the obstacles to seeking immunization services.

*It breaks my heart whenever my children ask me for anything and I cannot give them.*

[A caregiver from Kachia]

In rural or underserved locations with inadequate infrastructure and resources, caregivers highlighted difficulties with health care facility availability and accessibility. To address systemic problems and enhance vulnerable groups’ access to health care, these obstacles highlight the necessity for focused treatments.

*I trek for thirty minutes to get to the PHC.*

[A caregiver from Kachia]

The study found that while caregivers drew on a range of sources for health education and information sharing, health professionals, traditional leaders, and religious figures in the community made significant contributions. Providing accurate and trustworthy information regarding health benefits, preventive measures, and available health care services is a critical responsibility of health workers. Health workers supported caregivers in better recognizing their own health needs and in seeking appropriate care when needed through one-on-one contacts, counseling sessions, and community outreach programs.

Besides health care professionals, traditional and religious leaders provided valuable counsel and assistance to caregivers. These leaders were frequently regarded as reliable sources of information within the community. These leaders used their power and influence to encourage adherence to advised health practices, debunk myths, and promote positive health behaviors. Traditional and religious leaders were crucial in promoting health education messaging and influencing caregivers’ attitudes and behaviors about health care by introducing health-related themes into sermons, community meetings, and cultural activities.

#### Family Dynamics and Decision-Making

The study revealed significant variations in marital dynamics among caregivers, highlighting differences in spousal support, extramarital affairs, and decision-making autonomy across communities. In some communities, caregivers reported experiencing strong spousal support, where husbands actively participated in household responsibilities, provided emotional and financial assistance, and demonstrated a collaborative approach to decision-making. However, in contrast, caregivers in other communities described strained marital relationships characterized by limited support from their spouses, instances of extramarital affairs, and unequal decision-making power. These disparities underscored the complex interplay of cultural norms, socioeconomic factors, and individual dynamics influencing marital relationships within the studied communities.

*I don’t want my children to live the kind of life I’m living now; I want to send them to school so that they can get good jobs.*

[A caregiver from Soba]

Associations emerged as integral components of the social fabric within the communities, offering caregivers opportunities for socialization, knowledge exchange, and collective support. These associations provided a platform for women to come together, share experiences, and offer mutual assistance in navigating various challenges they faced. Through regular meetings, group discussions, and collaborative activities, caregivers benefited from the wisdom and support of their peers, fostering a sense of solidarity and empowerment within the community. Additionally, female associations served as conduits for disseminating health education messages, promoting healthy behaviors, and mobilizing community action to address common concerns. By leveraging these support networks, caregivers were able to access valuable resources, build social capital, and strengthen their resilience in the face of adversity.

#### Future Aspirations and Community Development

As a way to improve their families’ standard of living and financial standing, caregivers expressed a significant desire for economic empowerment, according to the study’s findings. Aspired to improve their financial well-being and create a sustainable revenue stream, caregivers expressed a desire for entrepreneurial options and vocational training. Caregivers expressed a strong desire to learn new skills related to tailoring, catering, and entrepreneurship to start profitable businesses through interviews and participatory discussions. The caregivers’ strong will and tenacity to escape the cycle of poverty and provide better possibilities for their families and themselves were highlighted by these ambitious goals. To help caregivers reach their full potential, promote their financial independence, and advance community development, stakeholders should fund economic empowerment programs.

*I want to learn a trade but my husband has to give me permission.*

[A caregiver from Soba]

The solutions provided by caregivers centered on targeted interventions aimed at raising the standard of living and resolving socioeconomic problems in the communities. Some of the key suggestions were constructing roads, supplying clean water, and installing electricity to bolster the infrastructure as a whole and encourage economic activity. To support small-scale enterprises and entrepreneurship, caregivers have also advocated for greater access to business financing, such as grants and loans for microfinance. Furthermore, a significant emphasis was placed on enhancing health care services to better treat prevalent health issues and improve community health outcomes. By placing a high focus on community development initiatives, stakeholders may promote social cohesion, facilitate sustainable growth, and help communities thrive in the face of financial challenges.

#### Vaccination Perceptions and Barriers

Participants expressed concerns over vaccine safety and practical challenges in accessing immunization services, underscoring the need for clear, culturally relevant education on vaccines. One caregiver from Soba explained that safety rumors are common in the community, remarking:

*Some people say that vaccines can harm our children, and that makes me fear taking them for immunization.*

Another caregiver from Kachia highlighted that widespread rumors about severe side effects further confuse caregivers, noting:

*I have heard rumours in my community about children developing serious sickness after getting vaccinated; it is hard to know what to believe.*

In addition, concerns over mixed messages from health providers were noted. A caregiver from Soba observed that inconsistent communication at the clinic contributed to ongoing confusion, stating:

*The clinic gives different messages about vaccines, which is confusing. I wish the information was clearer and more relevant to our culture.*

### Transition From 2-Arm to 3-Arm Intervention Design

During the ideation phase, discussions revealed that a 2-arm design, comprising only a control group (arm 1) and a financial empowerment group (arm 2), failed to fully capture the community’s multifaceted needs. While financial empowerment is essential, caregivers expressed that additional social support and education were needed to address vaccine hesitancy and improve health decision-making.

Based on community feedback and contextual insights, the study design was modified to include a third intervention arm focused on peer support and education (arm 3). This arm was conceptualized to offer monthly meetings where participants receive tailored immunization and child health education, led by trained community health workers, focusing on dispelling vaccine misinformation, addressing common vaccine-related concerns, and providing accurate information on vaccine safety and benefits. The sessions use culturally adapted tools, such as the *immunization hand tool* and a theme song, to reinforce key messages and enhance information retention. In addition to educational content, these meetings are designed to foster social support and skill-building, complementing the financial literacy training and microloan access provided to the financial empowerment group.

This integrated approach ensures that the intervention addresses both economic and social barriers, thereby maximizing the potential impact on RI uptake and overall community health. The revised 3-arm cluster randomized controlled trial (cRCT) design is summarized in Table 3.

**Table 3.** How the formative research informed the modification of the intervention design in the cluster randomized controlled trial.

Original study design	Challenge identified	Final study design
Number of arms		
Two-arm study (control and empowerment)	The 2-arm study was limited in contextualizing the issues related to empowerment at the community level Community members interpreted empowerment differently	Three-arm study
Factoral design		
Financial empowerment and control groups	Financial empowerment alone would not address community-level barriers such as cultural beliefs and perceptions The design did not take into account the role of peer support in encouraging health-seeking	Peer education and support Financial empowerment group Control group
Factors addressed		
Monitor the usage of the funds and how it relates to the uptake of immunization services	There was a disconnect between what the intervention would address and the stakeholders’ perception about the key drivers of immunization uptake	The new design addresses cooperative financing systems to foster collaboration and improve knowledge about immunization services using peer groups and support
Data collection		
Data to be collected at the facility level	The data collection source is not exhaustive enough to capture all the necessary indicators in the design	Immunization data from children Sociodemographic data from caregivers Attendance and participation in economic empowerment sessions, immunization, child nutrition, and hygiene knowledge sessions Loans and loan repayment from cooperative groups

## Discussion

### Principal Findings

This formative study was designed to inform the development of a culturally appropriate, women-centered cRCT aimed at identifying key sociocultural, economic, and healthcare-related barriers to RI uptake among caregivers, with the goal of increasing awareness and generating demand for RI services among economically disadvantaged populations. The study intricately integrated community engagement throughout the multilayered program development process, offering a unique perspective to guide the intervention. Specifically, the incorporation of HCD in the formative research phase has proven effective in promoting behavior change by ensuring that solutions are grounded in the lived experiences and needs of the target community [32]. The study findings revealed varying challenges such as financial constraints, limited health care access, sociocultural norms, and vaccine safety concerns, all affecting decision-making.

From the initial findings of the study, there was a need for an integrated intervention that addresses both financial and social barriers to improve RI uptake. These insights directly guided the design of a 3-arm cRCT using HCD within a CBPR to understand the specific needs and requirements for developing a culturally appropriate program to enhance mothers’ awareness, support, and economic empowerment, ultimately capable of improving uptake of immunization services and more effectively addressing the challenges of vaccine hesitancy.

### Addressing Vaccine Hesitancy Through Structured Communication and Leveraging Community Groups for Health Promotion and Behavior Change

One of the key themes that emerged as a persistent gap in RI uptake was perceptions and barriers to vaccination, which aligns with global evidence on vaccine hesitancy. Caregivers expressed fears about potential side effects of vaccines as well as misinformation such as rumors that vaccines are potentially harmful to children. This mirrors findings from low-resource settings where lack of trust in health systems exacerbates low immunization uptake [33]. The fears of caregivers about vaccines were further compounded by mixed messages from health care providers, and this emphasizes the need for a structured, culturally tailored communication as demonstrated in a similar context by Kaufman et al [34]. Community-led advocacy has been reported as a successful model to lower issues with vaccine hesitancy, where peer educators and religious leaders improved vaccine acceptance by bridging gaps between scientific evidence and local beliefs [35-37]. Using the voices of the community, this strategy builds community resilience and togetherness while also bolstering vaccine confidence [38-41]. Communities can set the path for higher vaccination rates and better public health outcomes by working together and leveraging local expertise and experiences. The core causes of vaccine hesitancy within certain cultural and social contexts can be effectively addressed by community-led lobbying [41-44]. Advocacy groups can help close the gap between scientific knowledge and community perceptions by customizing messaging to fit local conventions, beliefs, and languages [40,45]. In addition to fostering inclusivity, this culturally aware approach makes sure that no member of the community is left out.

The community’s source of immunization information is crucial in lowering vaccine hesitancy. When advocacy initiatives come from within the community, they are more effective. This grassroots strategy guarantees that the messaging is understood by the community while also fostering trust. A culture of trust and acceptance can be fostered by giving community members the authority to speak out in favor of vaccinations [46,47]. Advocacy campaigns sponsored by the community can cultivate a sense of empowerment and ownership among its members. People who take the initiative to spread factual information about vaccines engage in a proactive role in preserving the health and welfare of their community. This sense of ownership creates a sense of shared accountability for promoting immunization as a community endeavor and overcoming vaccine skepticism. Traditional and religious leaders also provide valuable counsel and assistance to caregivers. These leaders were frequently regarded as reliable sources of information within the community. These leaders use their power and influence to encourage adherence to advised health practices, debunk myths, and promote positive health behaviors. Traditional and religious leaders are crucial in promoting health education messaging and influencing caregivers’ attitudes and behaviors about health care by introducing health-related themes into sermons, community meetings, and cultural activities.

The importance of personalized information campaigns such as community mobilization is superior to general messaging in helping overcome vaccine hesitancy; this finding is consistent with other studies [48,49]. Community groups emerge as integral components of the social fabric within the communities, offering caregivers opportunities for socialization, knowledge exchange, and collective support. These groups provide a platform for women to come together, share experiences, and offer mutual assistance in navigating various challenges they face. Through regular meetings, group discussions, and collaborative activities, caregivers benefit from the wisdom and support of their peers, fostering a sense of solidarity and empowerment within the community. Female associations serve as conduits for disseminating health education messages, promoting healthy behaviors, and mobilizing community action to address common concerns. By leveraging these support groups, caregivers were able to access valuable resources, build social capital, and strengthen their resilience in the face of adversity.

Community groups also offer a forum for the dissemination of health education and information that is appropriate for a certain culture [50,51]. Caregivers can obtain accurate information about illness management, preventive health measures, and available health care services through workshops, educational sessions, and group discussions led by reputable community leaders or medical professionals. Health promotion initiatives become more effective when tailored to the cultural norms, values, and beliefs of the community. Adapting messaging to align with these cultural aspects enhances engagement and increases the likelihood of behavior change as cultural adaptations in health interventions aim to make services more linguistically appropriate, culturally competent, and safe, addressing disparities and improving outcomes for diverse populations [52-55]. Community groups present chances for lobbying and group action aimed at removing structural obstacles to health care use and access. Through the mobilization of caregivers as advocates for enhanced health care services, policies, and infrastructure, these groups can bring about significant changes at the community level that will benefit everyone’s health.

### Addressing Economic Barriers to Health Care Access Through Financial Empowerment and Women’s Autonomy in Health Decision-Making

Financial constraint was another theme identified, and this hindered caregivers from accessing health care services, including RI, as many caregivers expressed that limited household income restricted their ability to make health-related decisions for themselves and their children such as medical care, essential medications, and transportation to health facilities. These financial challenges mostly led to delays in seeking care and, in some cases, avoidance of health services. A significant number of women conveyed their desire to have more control over their financial resources, to secure a steady source of income, and to enhance their financial independence, enabling them to prioritize their health and that of their families and vocational training to improve their ability to prioritize family health needs. Our results corroborate studies that reported that out-of-pocket expenses for medications and transportation represent significant obstacles for families with limited financial capabilities. These costs can be unaffordable, forcing families to forgo needed medical care or make difficult choices about basic necessities [56-58], and this mirrors patterns observed in similar sub-Saharan African contexts [59].

It is important to state that financial empowerment plays a crucial role in empowering women to take charge of their health and well-being, ensuring better access to essential health care services and medications for both themselves and their families [60,61]. It is a crucial factor influencing women’s agency in health decision-making processes. Studies have shown that interventions promoting economic empowerment increase women’s involvement in making decisions about their own and their children’s well-being [62,63].

Access to economic power provides women with resources, opportunities, and decision-making authority, enabling them to prioritize health, access health care services, and contribute to the overall well-being of their households [64]. This also influences children’s immunization, as women are more likely to prioritize vaccinations for their children when they have the financial means and autonomy to make health-related decisions [65]. This connection between financial empowerment and women’s agency in health decisions demonstrates the importance of addressing economic disparities to enhance women’s health outcomes and promote gender equality. By empowering women financially, stakeholders can promote greater autonomy in health decision-making, enhance health care use rates, and improve health outcomes for women and their families.

### Addressing Sociocultural Barriers to Immunization Through Women Empowerment and Navigating Religious Influences in Health Decision-Making

Sociocultural norms, influenced by religious diversity, influenced caregivers’ decision-making and their approach to health practices, which potentially can affect RI uptake. The varying levels of empowerment and autonomy among women affected their ability to make informed decisions regarding vaccination. In communities where women had limited decision-making power or faced resistance from male family members, immunization uptake was often hindered. These sociocultural factors, combined with misinformation and fear of vaccine side effects, contributed to low RI rates.

The observed influence of religious diversity on health practices aligns with previous studies documenting how faith-based beliefs shape vaccination attitudes across different cultural settings [66]. In particular, our findings resonate with studies from similar settings where religious interpretations of disease prevention have created both barriers and opportunities for immunization programs [66,67]. The gender dynamic observed in decision-making processes is a well-documented pattern in male-dominated societies, where male authority over health decisions usually results in lower child vaccination coverage [68,69]. This situation has been particularly well documented in Northern Nigeria, where studies have reported that spousal approval requirements can reduce immunization likelihood by up to 40% [70,71]. By implication, this can lead to a decrease in vaccine uptake, weaken herd immunity, and increase the risk of vaccine-preventable diseases.

Other identified barriers were inadequate availability of supplies and medications at health care facilities and poor infrastructure in these rural and underserved areas, which contribute to challenges in accessing immunization services. To address systemic problems and enhance vulnerable groups’ access to health care, there is a need for focused interventions. While caregivers leveraged a range of sources for health education and information sharing, it remains important for health workers to provide accurate and trustworthy guidance on health benefits, preventive measures, and available services to caregivers, as they play a crucial role in helping caregivers identify their health needs and seek appropriate care.

### Implications for the cRCT Study Design

The contextual findings in this formative research were instrumental in the thinking of how to modify the original 2-arm study which was identified to have several challenges, as standalone economic interventions often fail to address deep-rooted vaccine hesitancy. Financial empowerment alone would not address community-level barriers such as cultural beliefs and perceptions toward RI uptake. The design did not take into account the role of peer support in encouraging health-seeking behavior. The study uncovered a limitation in contextualizing the issues related to empowerment at the community level, as empowerment meant different things to the community members. To ensure we developed a culturally appropriate study, we modified our initial study design to incorporate a factorial design with 3 arms: peer support and education to tackle sociocultural barriers through trusted networks, financial empowerment to address economic barriers, and a control. This guaranteed that all the most relevant issues that were identified at the formulation phase were incorporated into the final study design.

### Limitations

This study demonstrates some key strengths that enhance its validity and potential impact. First, the use of HCD with a CBPR ensured that the research was deeply rooted in local realities, capturing nuanced sociocultural, economic, and structural barriers to RI. The study also prioritized cultural relevance and community ownership by engaging caregivers, religious leaders, and health workers in this formative phase, which are critical for intervention sustainability. Second, the transition from a 2-arm to a 3-arm cRCT design encouraged mixed insights with evidence from comparable settings, which strengthened the robustness of our findings and addressed gaps by providing interventions to bridge financial and sociocultural barriers, aligning with the need for a multicomponent strategy in gender-inequitable settings. Third, the study’s emphasis on culturally tailored communication and community-led advocacy reflects best practices in global health literature, where trust-building and behavioral change strategies have proven more effective. Finally, the research design explicitly addressed intersectional vulnerabilities, such as poverty, gender norms, and rural access and offers a replicable model for other low-resource settings with similar RI uptake challenges.

This formative work has certain limitations. One limitation of the study was the potential for response bias during data collection, as respondents may have been hesitant to provide candid responses due to unfamiliarity with the research team. Despite efforts to mitigate this bias through the involvement of local female community resource persons, some degree of reluctance or social desirability bias may have influenced the data. Additionally, the study’s focus on specific LGAs within Kaduna State may limit the generalizability of findings to other regions or populations with different sociocultural contexts. Furthermore, the study’s reliance on self-report data, particularly regarding income and daily activities, may have introduced inaccuracies or inconsistencies. Future research could use more diverse sampling methods and incorporate objective measures to enhance the robustness and applicability of findings.

### Conclusions

In conclusion, our formative research aimed to develop a culturally appropriate, women-centric cRCT study focused on enhancing RI service uptake among economically disadvantaged caregivers. Through community engagement using HCD, we gained valuable insights into barriers to immunization service use among mothers. Leveraging community groups for health promotion and advocacy emerged as a powerful strategy to combat vaccine hesitancy and improve public health outcomes. Community-led initiatives were indicated to foster trust, inclusivity, and empowerment, bridging gaps between scientific knowledge and community perceptions. Financial empowerment emerged as a pivotal factor in women’s ability to make health-related decisions and access health care services.

Enhancing economic opportunities for women not only promotes agency in health decision-making but also contributes to overall household well-being. Our findings highlight the importance of addressing gender-centered economic disparities to improve women’s health outcomes and promote gender equality. The sociocultural context significantly influences health care access and use, highlighting the need for targeted interventions. Religious diversity, early pregnancy, marriage, and financial constraints were identified as key determinants affecting health-seeking behaviors. Addressing these barriers requires tailored approaches that consider community norms, beliefs, and infrastructure challenges.

These findings have informed modifications to our study design, incorporating peer support, education, financial empowerment, and control arms in a factorial design. This ensures a holistic approach to address community-level barriers and enhance the cultural appropriateness of our intervention. Our formative research lays a solid foundation for a comprehensive cRCT study aimed at promoting immunization uptake and addressing health disparities among vulnerable populations.

## Abbreviations

CBPA: community-based participatory approach
CBPR: community-based participatory research
cRCT: cluster randomized controlled trial
FGDs: focus group discussions
GRISP: Global Routine Immunization Strategies and Practices
HCD: human-centered design
HCD: human-centered design
Hib: *Haemophilus influenzae* type b
KIIs: key informant interviews
LGAs: local government areas
LQAS: Lot Quality Assurance Sampling
PAPA: Performance Assessment for Performance Management
PHC: primary health center
RI: routine immunization
RIBS: Routine Immunization Buddy System
